# Alteration of tumor-associated macrophage subtypes mediated by KRT6A in pancreatic ductal adenocarcinoma

**DOI:** 10.18632/aging.104091

**Published:** 2020-11-18

**Authors:** Junfeng Zhang, Hui Sun, Songsong Liu, Wenjie Huang, Jianyou Gu, Zhiping Zhao, Huan Qin, Liwen Luo, Jiali Yang, Yongfei Fang, Jiayun Ge, Bing Ni, Huaizhi Wang

**Affiliations:** 1 Institute of Hepatopancreatobiliary Surgery, Chongqing General Hospital, University of Chinese Academy of Sciences, Chongqing 401120, P R China; 2 Department of Rheumatology, First Affiliated Hospital of Third Military Medical University, Chongqing 400038, P R China; 3 Institute of Hepatopancreatobiliary Surgery, Southwest Hospital, Army Medical University (Third Military Medical University), Chongqing 400038, P R China; 4 Department of Hepatobiliary Surgery, Zhujiang Hospital, Southern Medical University, Guangzhou 510280, Guangdong Province, P R China; 5 Department of Orthopedics, Xinqiao Hospital, Army Medical University (Third Military Medical University), Chongqing 400038, P R China; 6 Hepatopancreatobiliary Surgery Department, the Second Affiliated Hospital of Kunming Medical University, Kunming 650101, Yunnan Province, P R China; 7 Department of Pathophysiology, College of High Altitude Military Medicine, Third Military Medical University, Chongqing 400038, P R China; 8 Key Laboratory of Extreme Environmental Medicine, Ministry of Education of China, Chongqing 400038, P R China; 9 Key Laboratory of High Altitude Medicine, PLA, Chongqing 400038, P R China

**Keywords:** pancreatic ductal adenocarcinoma, tumor-associated macrophages, KRT6A, tumor immune microenvironment

## Abstract

Pancreatic ductal adenocarcinoma (PDAC) is severely affecting the health and lives of patients. Clarifying the composition and regulatory factors of tumor immune microenvironment (TIME) is helpful for the treatment of PDAC. We analyzed the unique TIMEs and gene expression patterns between PDAC and adjacent normal tissue (ANT) using Gene Expression Omnibus (GEO) to find new immunotherapy targets. The Cancer Genome Atlas (TCGA) datasets were used to elucidate the possible mechanism of which tumor-associated macrophages (TAMs) changed in PDAC. We found that the composition of TAMs subtypes, including M0, M1, and M2, was different between PDAC and ANT, which was validated in recently published single-cell RNA-seq data. Many immune cells interacted with each other to affect the TIME. There were many DEGs enriched in some pathways that could potentially change the immune cell composition. KRT6A was found to be a DEG between PDAC and ANT that overlapped with DEGs between the M0-high group and the M0-low group in TCGA datasets, and it might alter and regulate TAMs via a collection of genes including COL5A2, COL1A2, MIR3606, SPARC, and COL6A3. TAMs, which could be a target of immunotherapy, might be influenced by genes through KRT6A and indicate an undesirable prognosis in PDAC.

## INTRODUCTION

Pancreatic ductal adenocarcinoma (PDAC) is a highly aggressive cancer with the poorest prognosis among various cancer types [1, 2]. The mechanism of developing PDAC involves hereditary factors, environmental factors, individual differences, and perhaps even more important, immunological factors, which have been suggested to affect the proliferative and metastatic capabilities of tumor cells [3]. There is growing evidence that the tumor immune microenvironment (TIME), containing T cells, B cells, dendritic cells, macrophages, fibroblasts, and other immunocytes or immune molecules, could be the target of immunotherapy of tumors [4, 5]. For example, fibroblasts in PDAC are composed of several populations functioning to impact the immunologic tumor microenvironment [6].

Tumor-associated macrophages (TAMs), an important component of the TIME, are a kind of tumor-infiltrating cell derived from circulating peripheral blood [7]. Macrophages in the tumor microenvironment are often called TAMs and contain three phenotypes: M0, M1, and M2. It was previously believed that these TAMs played a pro-tumoral role [8]. However, recent research found that M1 macrophages have pro-inflammatory and anti-tumoral effects and are associated with a good prognosis in some cancers, whereas M2 macrophages are present in immunosuppressive states and have a pro-tumoral effect [9]. M0 macrophages are undifferentiated and nonpolarized subtypes and can potentially be polarized toward M1 or M2 macrophages, which is crucial for forming a TAM network in the TIME [10, 11]. A high density of TAMs was proved to be associated with poor survival rates in breast cancer [12]. TAMs, which are infiltrated more in colorectal cancer (CRC) [13], can stimulate the growth of tumor cells by altering extracellular matrix remodeling, tumor metabolism, angiogenesis, and the tumor microenvironment and may serve as a target for CRC treatment [14]. TAMs exhibit anti-tumoral properties in Sonic Hedgehog-related medulloblastoma by impairing tumor growth, in contrast to the pro-tumoral role played by TAMs in glioblastoma [15]. PD-1 expression by TAMs negatively correlates with phagocytic potency against tumor cells [16]. TAMs seem to affect the function of various tumor cells.

Moreover, there has been great progress in targeting TAMs as a form of immunotherapy for cancers. Because TAM infiltration is associated with poor patient outcomes, systematic and well-defined criteria for the evaluation of macrophage populations are required for practical TAM-targeting diagnostic and therapeutic strategies [17]. Selective targeting of TAMs via nanocarriers has proved to be beneficial in the treatment of cancer because TAMs display many upregulated surface proteins compared to non-TAMs [18, 19]. The multilayered relationship between cancer stem cells and TAMs potentially represents an innovative therapeutic target [20]. Targeting TAMs via CCL2/CCR2 signaling has been used as a therapeutic strategy against hepatocellular carcinoma [21]. In PDAC, TAMs have been exploited to select patients more likely to respond to the postsurgical adjuvant chemotherapy cyclophosphamide, which provides the basis for novel strategies aimed at re-educating macrophages in the context of cyclophosphamide [22]. This research suggests that TAMs could be potential therapeutic targets in PDAC. Therefore, in our article, we illustrate discrepant TIMEs between PDAC and adjacent normal tissue (ANT) and identify clusters of genes that facilitate alteration of TAMs.

## RESULTS

### Some immune cell types were different in frequency between normal tissue and pancreatic cancer

To unveil different characteristics between PDAC and normal tissues, we selected datasets based on three criteria: containing gene expression profile of both PDAC and non-tumor tissues; integrated and available gene expression profile of each dataset; a large sample size of each dataset as possible. As a result, GSE15471, GSE16515, GSE28735, GSE62165, and GSE62452 datasets were filtered and acquired from Gene Expression Omnibus (GEO) and were RMA normalized. With the help of the immune infiltration analysis tool CIBERSORT, the ratio of 22 immune cell types in five GEO datasets, including GSE15471, GSE16515, GSE28735, GSE62165 and, GSE62452, was acquired. Each dataset was divided into two groups according to their original experimental design, ANT and PDAC.

**Figure 1:**
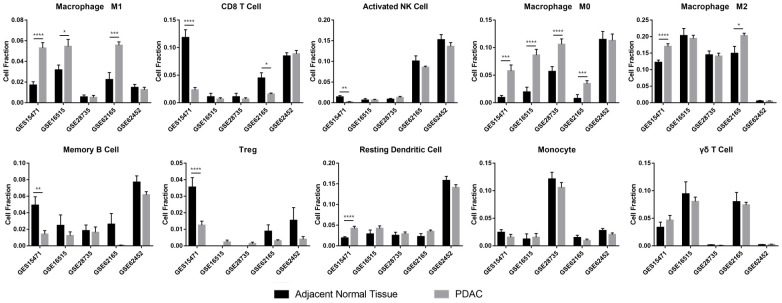
**Immune infiltration analysis of ANT and PDAC.** The immune infiltration analysis tool CIBERSORT was used to characterize 22 immune cell types in ANT and PDAC in five datasets (GSE15471, GSE16515, GSE28735, GSE62165 and GSE62452). *: p<0.05, **: p<0.01, ***: p<0.001, ****: p<0.0001.

Some immune cell types were significantly different in quantity between the two groups (Figure 1). Although accurate cell numbers could not be estimated directly, it seemed that the ratios of CD8^+^ T cells, activated NK cells, memory B cells, Tregs and resting dendritic cells were significantly different only in one or two datasets, which suggested the potential function of these cells in the PDAC TIME. However, the fraction of M0 macrophages was higher in PDAC than in ANT in four of the five datasets based on gene expression data of bulk tissues. Similarly, the fraction of M1 macrophages and M2 macrophages was also increased in PDAC compared with ANT in almost half of the datasets. These results suggested a critical role for macrophages in PDAC. Therefore, we took TAM subtypes as the research object in our research.

**Figure 2:**
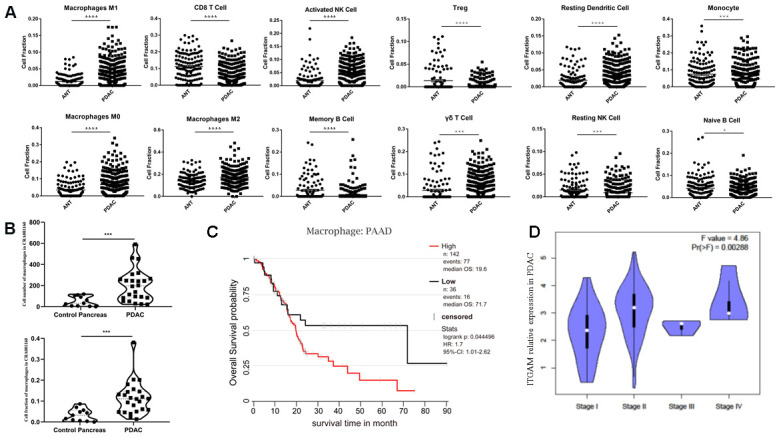
**Immune infiltration analysis of ANT and PDAC by integrating datasets.** (**A**) Immune infiltration analysis of ANT and PDAC by integrating five datasets including GSE15471, GSE16515, GSE28735, GSE62165, and GSE62452. (**B**) Cell number (upper) and cell fraction (lower) of macrophages in PDAC and normal pancreas according to the single-cell sequence dataset CRA001160. (**C**) Survival analysis of PAAD patients as influenced by the number of macrophages. (**D**) Violin plot of ITGAM expression in different stages of PDAC in patients from TCGA.

By integrating all five datasets, identical results were obtained that several immune cell types were distributed differently in ANT and PDAC, including M0, M1, and M2 macrophages (Figure 2A). In addition, we validated our results using recently published single-cell RNA-seq data [23], CRA001160, which was publicly available in the Genome Sequence Archive under project PRJCA001063 in China. The data showed an increased number and fraction of macrophages in PDAC compared to the control pancreas in CRA001160, which was the same as our result in 5 GEO datasets (Figure 2B).

Taken together, these results suggest a substantial change in the TIME in PDAC relative to ANT, which indicates its crucial role in the development of PDAC. Moreover, M1, M2 and especially M0 macrophages may play an unexpected key role in PDAC, and, we found that macrophages are indeed involved in the prognosis of PDAC (Figure 2C). The expression of the macrophage marker ITGAM seemed positively correlated with PDAC progression (Figure 2D). Additionally, multivariate analysis of ITGAM low and high expression PDAC patients in TCGA (n = 176) showed ITGAM expression (p = 0.032) and survival status (p = 0.046) were significantly related with clinical stage, whereas gender (p = 0.323) and age (p = 0.662) were not significant (Supplementary Table 1).

### Many immune cells interact with each other to comprehensively affect the PDAC TIME

**Figure 3:**
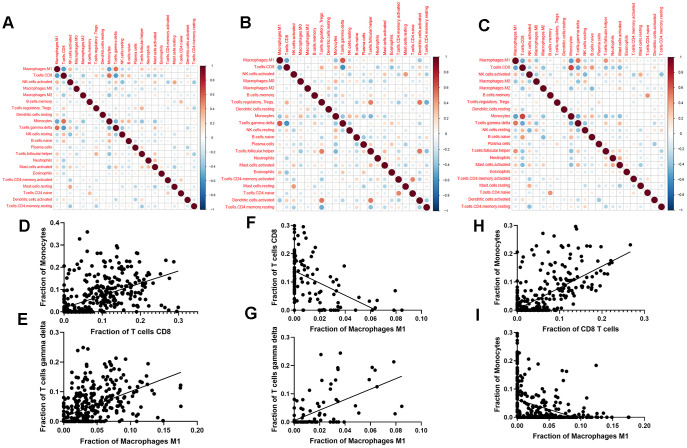
**Correlation between different immune cell types.** (**A**–**C**) Correlation between different immune cell types in the combined data of PDAC and ANT (**A**), ANT (**B**) and PDAC (**C**). (**D**–**I**) Scatter diagrams of immune cell fraction in PDAC and ANT (**D**, **E**), ANT (**F**, **G**) and PDAC (**H**, **I**).

We next evaluated the correlations among different immune cell types in PDAC + ANT (Figure 3A), ANT (Figure 3B), and PDAC (Figure 3C), respectively. There were completely different correlations among different immune cell types. Specifically, monocytes had a positive correlation with CD8^+^ T cells (Figure 3D) and gamma delta T cells had a positive correlation with M1 macrophages (Figure 3E) in PDAC+ANT group. M1 macrophages had a positive correlation with gamma delta T cells (Figure 3G) but a negative correlation with CD8^+^ T cells (Figure 3F) in the ANT group. In addition, monocytes had a positive correlation with CD8^+^ T cells (Figure 3H) but a negative correlation with M1 macrophages in the PDAC group (Figure 3I). These results suggested that immune cells might interact with particular cell types to comprehensively influence the TIME in PDAC.

### DEGs between ANT and PDAC were identified

**Figure 4:**
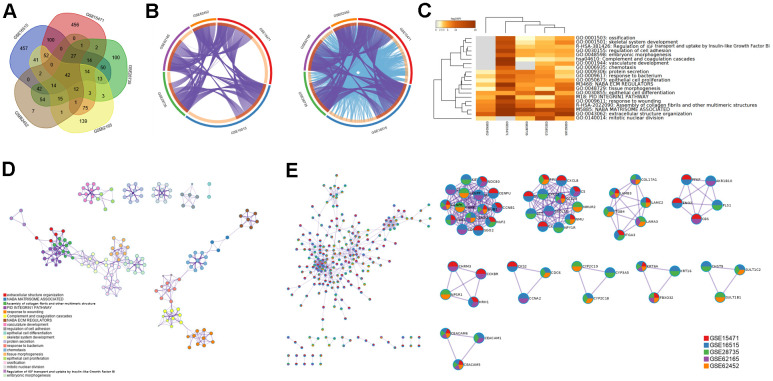
**Overlapped DEGs and pathways between PDAC and ANT in five datasets.** (**A**) Venn diagram of overlapped DEGs between PDAC and ANT in five datasets. (**B**) Overlapped genes and ontology terms in each dataset analyzed by Metascape. (**C**) Pathway enrichment analysis of DEGs in each dataset. (**D**) Pathway-pathway interaction network diagram. (**E**) Protein-protein interaction network among five datasets.

The number of overlapping DEGs between ANT and PDAC was 42 (Figure 4A). A portion of DEGs in every GEO dataset overlapped with other datasets and fell into the same ontology term (Figure 4B), which suggested strong consistency between different studies and individuals.

Pathway enrichment analysis of DEGs showed that regulation of insulin-like growth factor (IGF) transport and uptake by insulin-like growth factor Bi was enriched in 4 of 5 data sets (Figure 4C), and these pathways have been proven to affect macrophages [24, 25]. In addition, we found much connection among pathways (Figure 4D) and identified the PPI network of MCODE components (Figure 4E), which might work synergistically to affect the TIME.

**Figure 5:**
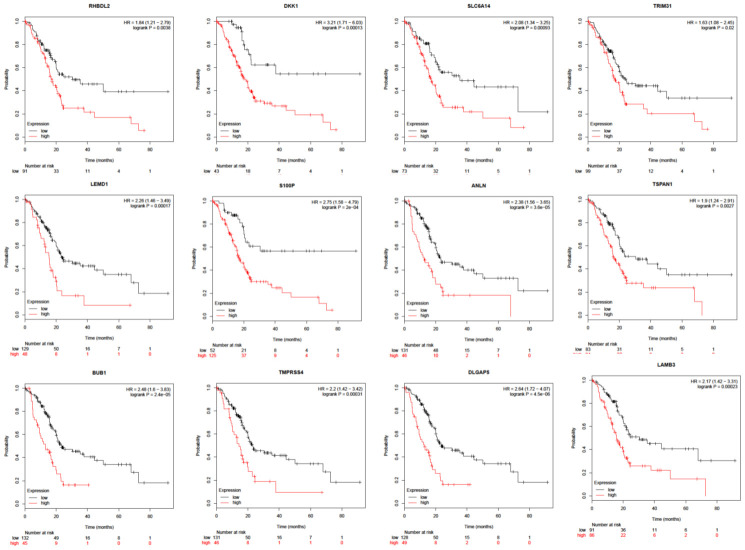
**Survival analysis of overlapped DEGs between PDAC and ANT in five datasets.**

The majority of identified DEGs were effective in evaluating prognosis using the Kaplan-Meier plotter (Figure 5).

### M0 macrophages were correlated with multiple immune cells

**Figure 6:**
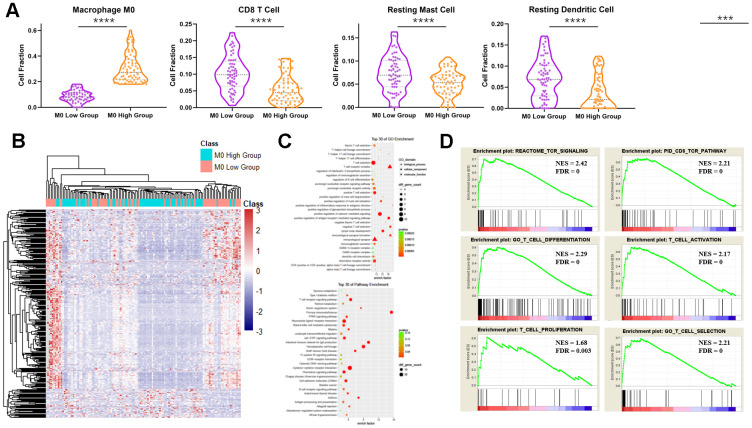
**The ratio of immune cells and gene expression and pathway patterns as a consequence of M0 macrophage numbers in PDAC.** (**A**) Numbers of immune cells in the M0-high group and M0-low group in PDAC according to TCGA data. ***: p<0.001, ****: p<0.0001. (**B**) Heatmap of the gene expression patterns of the M0-high group and the M0-low group in PDAC. (**C**) GO (upper) and KEGG (lower) pathway enrichment analyses of DEGs between the M0-high group and the M0-low group. (**D**) GSEA between the M0-high group and the M0-low group.

By dividing the TCGA PDAC mRNA-seq data into two groups, the M0-low group and the M0-high group, with the help of CIBERSORT, we obtained a number of immune cell types related to M0 macrophages in PDAC (Figure 6A). The numbers of CD8 T cells, resting mast cells, resting dendritic cells and M1 macrophages were significantly less in the M0-high group than in the M0-low group. M0 macrophages seemed to correlate with multiple immune cells.

DEGs between the two groups showed a unique expression pattern (Figure 6B), which perhaps led to different numbers of other immune cells. GO and KEGG pathway enrichment analysis showed that many T cell-related pathways were enriched (Figure 6C). A collection of T cell selection- and T helper cell lineage commitment-related pathways were enriched, which indicated that adaptive immunity might be involved in TAM modification. Identical conclusions were obtained using GSEA (Figure 6D). The TCR signal (NES = 2.42, FDR = 0), CD8 TCR (NES = 2.21, FDR = 0), T cell differentiation (NES = 2.29, FDR = 0), T cell activation (NES = 2.17, FDR = 0), T cell proliferation (NES = 1.68, FDR = 0.003) and T cell selection (NES = 2.21, FDR = 0) pathways were all enriched in the M0-high group, demonstrating that TAMs have a critical effect on adaptive immunity in PDAC.

### KRT6A influenced the frequency of TAMs in PDAC

**Figure 7:**
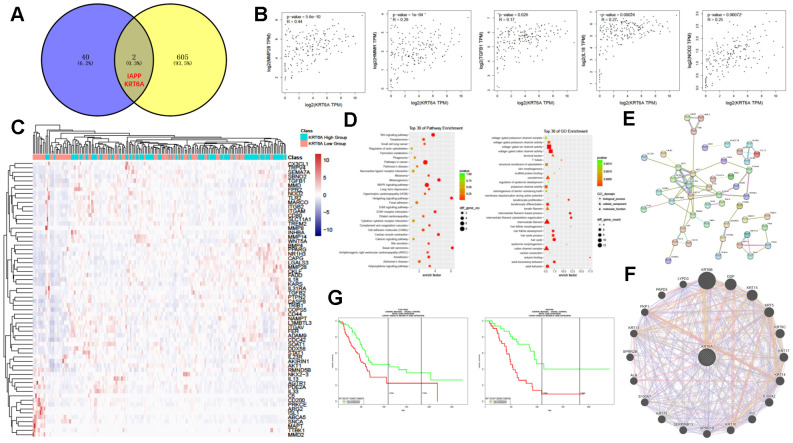
**KRT6A may participate in regulating TAMs in PDAC.** (**A**) Venn diagram of common DEGs between PDAC and ANT and between the M0-high group and the M0-low group. (**B**) Correlation of gene expression between KRT6A and various TAM-related genes in PDAC. (**C**) Heatmap of TAM-related gene expression patterns of the KRT6A-high group and KRT6A-low group in PDAC. (**D**) KEGG (left) and GO (right) pathway enrichment analyses of DEGs between the KRT6A-high group and the KRT6A-low group. (**E**, **F**) Protein-protein interaction network related to KRT6A using STRING (**E**) and GeneMANIA (**F**). (**G**) Survival analysis of the KRT6A-high group and the KRT6A-low group in TCGA PAAD data (left) and GSE57495 (right).

Next, we investigated the mechanism of TAM alteration in PDAC. We intersected the DEGs between the M0-low group and the M0-high group in TCGA and the DEGs between ANT and PDAC in the above 5 GEO datasets to find the potential mechanism that increases the fraction of macrophages in tumor other than normal tissues. The threshold of DEGs between the M0-low group and the M0-high group in TCGA is FC > 2 and p < 0.05. Therefore, we firstly filtered significant DEGs between PDAC and ANT groups, we then intersected these DEGs with DEGs between the M0-low group and the M0-high group to find potential genes regulating tumor-associated macrophages. Finally, two genes, IAPP and KRT6A, were obtained by intersecting the DEGs between the M0-low group and the M0-high group in TCGA and the DEGs between ANT and PDAC in the above 5 GEO datasets (Figure 7A). IAPP has been proven to play a role in the activation of macrophages through IL-1 [26], and inflammatory cascades are triggered by the uptake of IAPP aggregates by macrophages [27] in type 2 diabetes. However, the other gene, KRT6A, had not been reported to influence the frequency of TAMs. Consequently, our subsequent investigation focused on the particular mechanism by which KRT6A affected TAMs in PDAC.

The correlation analysis showed that KRT6A was highly correlated with macrophage-related genes, including MMP28, HMMR, TGFB1, IL-18, and NOD2 (Figure 7B). The KRT6A-low group and the KRT6A-high group showed special macrophage-related gene expression patterns (Figure 7C). As expected, GO and KEGG pathway enrichment analysis showed that several cancer-related pathways were enriched (Figure 7D). However, some of the pathways that were proved to potentially affect the polarization or activation of macrophages, including MAPK signaling [28] and Wnt signaling [29], were substantially enriched in the KRT6A-high group, which provided indirect evidence of modification of TAMs by KRT6A. Interaction network analysis using the STRING database (Figure 7E) and GeneMANIA (Figure 7F) indicated that KRT6A might affect TAMs through different kinds of genes, some of which had been reported to have an effect on macrophages, such as different S100 proteins (S100A2, S100A7, and S100A4) [30] and PI3 [31]. Notably, KRT6A expression was clearly related to the prognosis of PDAC in distinct datasets (Figure 7G).

**Figure 8:**
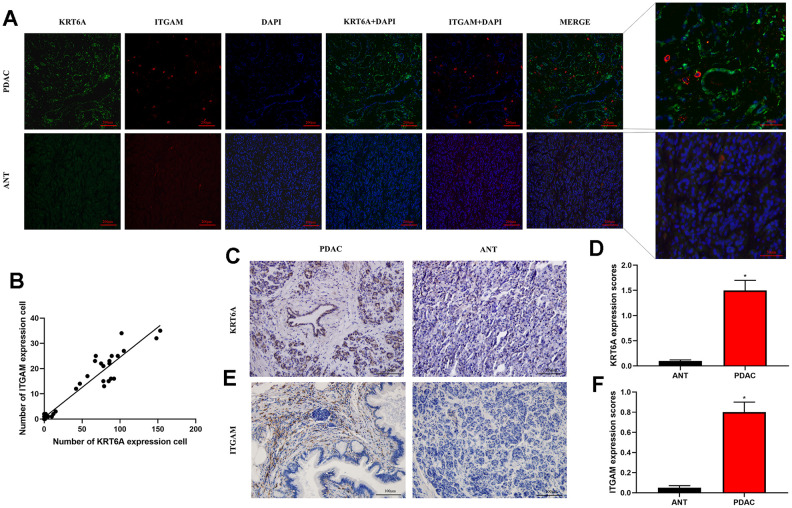
**Immunofluorescence and immunohistochemistry (IHC) staining by integrating KRT6A and ITGAM expression.** (**A**) Immunofluorescence staining of KRT6A (green) and ITGAM (red) in PDAC and ANT frozen tissue sections (100×). (**B**) Pearson's correlation of KRT6A and ITGAM expressing cells in PDAC and ANT. (**C**–**F**) IHC staining of KRT6A (c) and ITGAM (e) in PDAC and ANT (200×). Column charts were shown in (**D**) for KRT6A and (**F**) for ITGAM. *p<0.001.

Using immunofluorescence staining by integrating KRT6A and ITGAM expression and captured by a confocal microscope, we found that KRT6A and ITGAM were highly expressed in PDAC compared with ANT (Figure 8A). In addition, KRT6A staining seemed to be more pronounced near TAMs. There was high correlation between KRT6A expressing cells and TAMs (Pearson's correlation coefficient = 0.95, p < 0.001) ((Figure 8B). High expression of KRT6A and ITGAM in PDAC other than ANT was verified using immunohistochemistry (IHC). These results coordinated with the hypothesis above that KRT6A may play a role in regulating TAMs in PDAC.

### KRT6A regulated TAMs by a collection of genes

**Figure 9:**
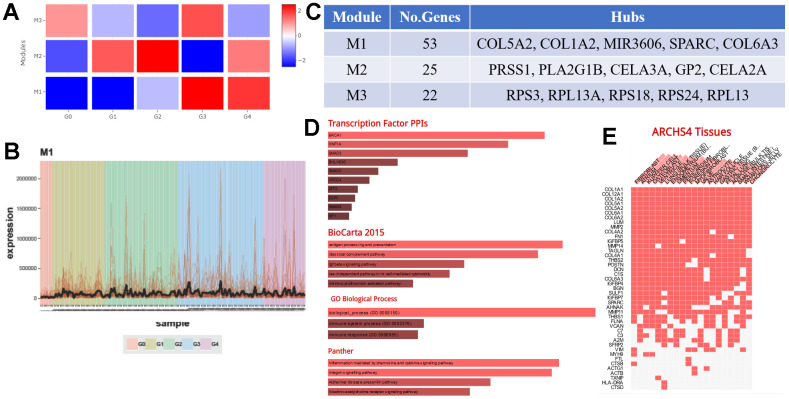
**The molecular mechanism by which KRT6A regulates TAMs.** (**A**) CEMiTool was used to find gene modules affecting TAMs through KRT6A by dividing TCGA PAAD mRNA-seq data into five groups by the level of KRT6A expression: Grade 0 (G0), Grade 1 (G1), Grade 2 (G2), Grade 3 (G3), and Grade 4 (G4), 6A expression where G0 expressed the lowest amounts of KRT6A (average RPKM = 0) and G4 expressed the highest amounts of KRT6A (average RPKM = 193.24). (**B**) Expression of a gene module (Module 1) in each PDAC patient. (**C**) Major genes and the number of genes in each module. (**D**) Transcription factor PPI network and pathway enrichment of genes in Module 1. (**E**) Cell type enrichment of genes in Module 1.

To identify genes the genes KRT6A targets to influence TAMs, we divided TCGA PAAD mRNA-seq data into five groups, Grade 0 (G0), Grade 1 (G1), Grade 2 (G2), Grade 3 (G3), and Grade 4 (G4), by the level of KRT6A expression, where G0 expressed the lowest amount of KRT6A (average RPKM = 0) and G4 expressed the highest amount of KRT6A (average RPKM = 193.24). CEMiTool illustrated those genes that were correlated with changes in KRT6A expression, which are potential key factors for regulating TAMs. The expression changes in one module of genes (Module 1) were perfectly consistent with KRT6A expression (Figure 9A, 9B). Major genes in Module 1 included COL5A2, COL1A2, MIR3606, SPARC, and COL6A3 (Figure 9C).

Several transcription factors and pathways enriched in Module 1 may participate in altering TAMs (Figure 9D). In addition, Module 1 genes were involved in a variety of immune-related pathways and enriched in a variety of cell types, including Kupffer cells, a kind of macrophage in the liver (Figure 9E), demonstrating the potential role of Module 1 genes in macrophages.

**Figure 10:**
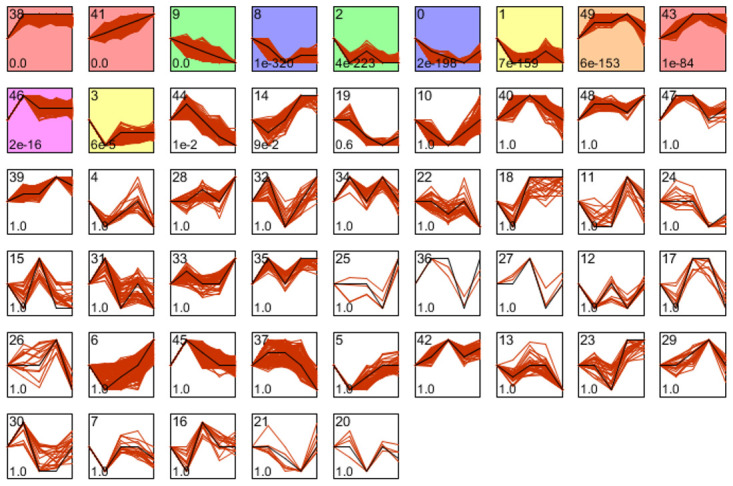
**Genes that were expressed similarly to KRT6A in PDAC were identified using the STEM tool.**

Finally, the Short Time-series Expression Miner (STEM) was used to cluster and visualize genes from G0 to G4 that had the same temporal expression patterns (Figure 10). Clusters 38 (MAML2, IL-18, MIR4785, ZFP36L1, LOC100506403, NCK1, KLF7, SUMO3, ARPC2, etc.) and 41 (ANXA8L1, ANXA8, KRT7, AHNAK2, MYOF, TLDC1, PTPRU, CDA, NOD2, TRIM29, etc.) exhibited a significant and obvious upward gene expression tendency, whereas cluster 9 (SLAIN1, PGPEP1, FAM189A2, RWDD2A, MPC2, RAI2, BTNL9, PRPSAP2, MTERF2, C22ORF39, etc.) exhibited a significant downward trend. Thus, we employed a novel method to identify the potential processes by which KRT6A affects TAMs in PDAC.

## DISCUSSION

Our research has investigated the tumor immune microenvironments (TIMEs) and gene expression patterns between pancreatic ductal adenocarcinoma (PDAC) and adjacent normal tissue (ANT) and found that several immune cell types, including M0, M1, and M2 macrophages, which include tumor-associated macrophages (TAMs), and many genes showed diverse expression patterns that were significantly correlated with the prognosis of PDAC. Then, we studied the underlying mechanism that altered TAMs and identified T cell-related pathways that were relevant to TAMs through pathway enrichment analysis. Subsequently, KRT6A was identified to affect TAMs through diverse proteins and pathways. Finally, using CEMiTool and STEM, we found that a collection of genes might participate in modifying TAMs through KRT6A in PDAC.

TAMs integrate anti-tumor activity (M1 macrophages) and pro-tumor activity (M2 macrophages) and have a strong effect on cancer eradication in PDAC [32]. TAMs have been proven to influence CD8 T cell-mediated tumor suppression to inhibit tumor cell invasion, metastasis, and desmoplasia through PI3Kγ in PDAC [33], which presents a novel therapeutic target for treating PDAC. In addition, M2 macrophage infiltration into the stroma is an independent prognostic factor for PDAC patients [34]. Consistent with these results, we found a completely distinct TIME in PDAC compared with that in ANT, which consisted of significantly higher frequency of TAM subtypes, including M0, M1, and M2 macrophages. Similarly, we found that TAMs had an effect on patient survival in PDAC.

Subsequently, we considered the cause of TAM alteration, focusing on immature macrophages and M0 macrophages in PDAC. Macrophages in cancer have been reported to activate or inhibit T cells based on macrophage phenotype, costimulatory molecules, and cytokine secretion and can significantly alter T cell activation and effector function [35], findings that are identical to our results. Apart from T cell activation, our results show that the TCR signal, CD8 TCR, T cell differentiation, T cell proliferation, and T cell selection pathways are all enriched in PDAC patients with high numbers of M0 macrophages. Our work implies that TAMs may have more contact, directly or indirectly, with innate immunity and with T cell-mediated immunity. TAMs may affect tumorigenesis, proliferation, and metastasis by transforming the features of T cells, resulting in compensatory activity against tumors.

By overlapping the DEGs between the M0-low group and the M0-high group in TCGA with the DEGs between ANT and PDAC, we acquired the potential regulatory genes of TAMs, IAPP, and KRT6A. KRT6A is related to cell invasion and metastasis of nasopharyngeal carcinoma via the β-catenin cascade [36]. The KRT6A-positive subset of mammary epithelial cells can be induced to form cancer by ErbB2 [37]. These findings suggest a substantial role of KRT6A across cancers. Although there is a lack of direct evidence that correlates with TAMs, KRT6A has been found to play a role in producing AMPs for innate immune defense against infection [38], which may indirectly affect macrophages. Our results show a positive correlation between KRT6A and TAM signature genes, as well as proteins and pathways that participate in regulating TAMs. Taken together, these results suggest that KRT6A is a novel potential therapeutic target aimed at TAMs during immunotherapy in PDAC.

We conclude that KRT6A alters TAMs by a collection of genes that have similar expression patterns to that of KRT6A. Several previously unreported genes clusters were identified using CEMiTool and STEM. Our hypothesis is that genes which regulate KRT6A will share an expression pattern with KRT6A. The genes that we identified in Module 1 were substantially enriched in antigen processing and presentation, immune system processes, the immune response, inflammation mediated by chemokine and cytokine signaling pathways, and especially the activities of Kupffer cells, a type of macrophage. In addition, some of the transcription factor that can influence TAMs were enriched, such as SMAD3 [39, 40], SMAD2 [41], SMAD4 [42], SP1 [43], BRCA1 [44]. This evidence strongly implies that the genes we identified could play a collective role in TAM activities through KRT6A.

In addition to the diverse immune cell patterns seen in PDAC, there are a group of genes whose expression was distinct in PDAC from that seen in ANT. These genes are enriched in tumor progression-related pathways, such as the regulation of cell adhesion, vasculature development, NABA ECM regulators, epithelial cell proliferation, and epithelial cell differentiation. Most of the identified genes are significantly correlated with the prognosis of PDAC, implying crucial roles of these genes in the development of pancreatic tumor cells, and these genes may be potential therapeutic targets in PDAC.

Our research found a unique TIME in PDAC that contains more TAMs than are seen in ANT. We proposed a unique process to identify the mechanisms by which KRT6A affects TAMs. The main aim of our study is to predict the potential key components of TIME in PDAC, which were further checked in PDAC tissues and ANT by immunofluorescence and IHC assays. However, the data in this study have not clarified the actual TIME in PDAC thoroughly, which needs future extensive experimental investigation.

## MATERIALS AND METHODS

### TCGA and GEO data acquisition and DEG filtering

The Cancer Genome Atlas (TCGA) pancreatic adenocarcinoma (PAAD) RNA-sequence data were acquired from Xena (https://xenabrowser.net/). In order to unveil differences characteristics between PDAC and normal tissues, we selected datasets basing on three criteria: containing gene expression profile of both PDAC and non-tumor tissues; integrated and available gene expression profile of each dataset; a large sample size of each dataset as possible. As a result, GSE15471, GSE16515, GSE28735, GSE62165, and GSE62452 datasets were filtered and acquired from GEO and were RMA normalized. Differentially expressed genes (DEGs) were obtained when fold change (FC) > 1.2 and p < 0.05. The FC of GSE62165 datasets was set to 1.5 because there were extensive DEGs in that group (approximately 3,098 genes in total) compared with the numbers of DEGs seen in other datasets when the FC = 1.2, which disturbed the equilibrium of all the data.

### Immune infiltration analysis

Immune infiltration of PDAC and ANT was performed using R 3.3.2 and the *CIBERSORT* package with 1,000 permutations. Samples whose CIBERSORT p-values were higher than 0.05 were excluded. The relative contents of 22 types of immune cells in PDAC and ANT were obtained, including B memory cells, naïve B cells, activated dendritic cells, resting dendritic cells, eosinophils, M0 macrophages, M1 macrophages, M2 macrophages, activated mast cells, resting mast cells, monocytes, neutrophils, activated NK cells, resting NK cells, plasma cells, activated memory CD4 T cells, resting memory CD4 T cells CD4, naïve CD4 T cells, CD8 T cells, T follicular helper cells, gamma delta T cells, and regulatory T cells (Tregs).

Recently published single-cell RNA-seq data, CRA001160, was downloaded from ftp://download.big.ac.cn/gsa/CRA001160 in National Genomics Data Center in China, to calculate the number and fraction of TAMs in PDAC and control pancreas tissues.

### Correlation analysis

Correlation between different immune cell types was calculated and visualized using the R package *corrplot*. Correlation between KRT6A gene expression and other macrophage-related genes was obtained using GEPIA (gepia.cancer-pku.cn).

### Survival analysis

Kaplan-Meier survival analysis and plotting of genes were performed on the Kaplan-Meier plotter (http://kmplot.com/analysis/) using pancreatic ductal adenocarcinoma mRNA datasets[45]. The Kaplan-Meier plotter is a web server for meta-analysis-based discovery and validation of survival biomarkers. Its mRNA subsystems include 54k genes from 21 cancer types. Gene expression data and relapse-free and overall survival information were downloaded from GEO, EGA, and TCGA. Additionally, survival analysis of patients with high macrophage amount and patients with low macrophage amount was performed via The Cancer Immunome Atlas (https://tcia.at/home) using the TCGA PAAD dataset.

### Pathway enrichment analysis

Pathway enrichment analysis of the key DEGs in our research was performed using Gene Ontology (GO) and Kyoto Encyclopedia of Genes and Genomes (KEGG) databases. The Metascape tool was used to find network relationships between DEGs (http://metascape.org/gp/index.html#/main/step1), while DEGs of GSE15471, GSE16515, GSE28735, GSE62165, and GSE62452 were uploaded.

### Gene Set Enrichment Analysis (GSEA)

GSEA was performed using GSEA 3.0 software. The gene sets mentioned in the article were downloaded using MSigDB (http://software.broadinstitute.org/gsea/index.jsp). The number of permutations was set to 1,000.

### Protein-protein interaction (PPI) network construction

A PPI network of KRT6A was constructed and visualized using STRING (https://string-db.org/cgi/input.pl) and GeneMANIA (http://genemania.org/).

### CEMiTool and Short Time-series Expression Miner (STEM)

CEMiTool (https://cemitool.sysbio.tools) and STEM software were used to find genes that had similar expression patterns to KRT6A in PDAC. TCGA PAAD RNA-seq data were grouped by expression of KRT6A and uploaded into CEMiTool and STEM software.

### Immunofluorescence staining

For immunofluorescence staining, frozen tissue section samples of PDAC and ANT were incubated with a mixture of rabbit anti-ITGAM (Proteintech; 1:50) and mouse anti-KRT6A (Proteintech; 1:50) antibodies overnight at 4°C after dewaxing, hydrating, antigen retrieval and blocking, followed by incubation with a mixture of Alexa Fluor 488 or Alexa Fluor 555 fluorescence-conjugated secondary antibodies (Abcam; 1: 200) for 1 hour and DAPI for 10 minutes. After each step, the specimens were rinsed three times with PBS for 5 minutes. The double-stained images were examined with an Olympus microscope and an Olympus FV1000 Confocal microscope.

### Immunohistochemistry (IHC) staining

Paraffin sections of PDAC tissue and ANT tissue were used for IHC staining. Sections were stained with primary antibodies against KRT6A (1:200, Proteintech Group, Chicago, IL, USA) and ITGAM (1:200, Proteintech Group, Chicago, IL, USA) according to the product manual after routine steps. Phosphate-buffered saline (PBS) was used as a control.

The scoring of positive immunoreactivity was performed. The intensity was classified as 0, 1+, 2+, and 3+, denoting no, weak, moderate, and strong staining, respectively. The distribution of staining was referred to as the percentage of positive tumor cells (0% to 100%). The final KRT6A and ITGAM expression scores were obtained by multiplying the two variables together.

## Supplementary Material

Supplementary Table 1
