# Supplementary material for: Alteration of tumor-associated macrophage subtypes mediated by KRT6A in pancreatic ductal adenocarcinoma
Source: Aging (Albany NY). 2020 Nov 18;12(22):23217–32. doi: 10.18632/aging.104091 (PMC7746340; doi:10.18632/aging.104091)
Supplement: Supplementary Table 1 [file aging-12-104091-s001..pdf]

SUPPLEMENTARY TABLE

Supplementary Table 1. Multivariate logistic regression analyzing of clinical stage of PDAC patients in TCGA in relation to ITGAM expression, gender, age and survival status.

Variables	OR	%95 CI	p-Value[a]
ITGAM expression (low / high)	2.530	1.084-5.908	0.032
Gender (male / female)	0.661	0.292-1.500	0.323
Age (≤55 / >55)	1.239	0.473-3.245	0.662
Survival status (alive / dead)	2.334	1.017-5.358	0.046

[a]Wald test for logistic regression.
